# Imaging of Cardiac Device-Related Infection

**DOI:** 10.3389/fcvm.2021.729786

**Published:** 2021-08-24

**Authors:** Jose Aguilera, Erika Hutt, Wael A. Jaber

**Affiliations:** Department of Cardiovascular Medicine, Cleveland Clinic Foundation, Cleveland, OH, United States

**Keywords:** cardiac device, cardiac implantable electronic devices, device infection, endocarditis, transesophageal echocardiogram, positron emission tomography, computed tomography, left ventricular assist devices

## Abstract

Cardiac devices are frequently used in different cardiovascular conditions for the purpose of morbidity or mortality prevention. These include cardiac implantable electronic devices (CIED) like permanent pacemakers and implantable cardiac defibrillators, ventricular assistance devices (VADs), left atrial appendage occlusion (LAAO) devices like the Watchman™, atrial and ventricular septal occluders like the Amplatzer™, among others. In the past years, there has been an increase in the development of these devices as a result of a rise in the number of indications for implantation, paired with the aging and more medically complex patient population. This has led to an increase in the incidence of cardiac device-related infections, one of the most feared and serious complications which is associated with significant morbidity, mortality and financial burden. Accurate diagnosis of cardiac device-related infections is essential given the management implications which often involve removal of the infected device, removal of other prosthetic material and long-term antimicrobial therapy. Clinical and laboratory data are useful diagnostic tools but multimodality imaging is often necessary. The recently published 2020 European Heart Rhythm Association International Consensus document, which is endorsed by many expert societies, has recommended the use of multimodality imaging for the diagnosis of CIED infections. (1) This allows better disease characterization by identifying abnormal fluid collections and guiding aspiration for both diagnostic and therapeutic purposes (i.e. soft tissue ultrasound and computed tomography), evaluation for local extent of disease (i.e. transesophageal echocardiogram to evaluate for concomitant infective endocarditis), embolic manifestation of disease (i.e. computed tomography and magnetic resonance imaging) and metabolic tissue characterization (positron emission tomography and tagged white blood cell scan). (2) In addition, computed tomography (CT) allows for pre-procedural planning which has shown to be associated with better procedural outcomes.

## Introduction

Cardiac devices are frequently used in different cardiovascular conditions for the purpose of morbidity or mortality prevention. These include cardiac implantable electronic devices (CIED) like permanent pacemakers and implantable cardiac defibrillators, ventricular assistance devices (VADs), left atrial appendage occlusion (LAAO) devices like the Watchman™, atrial and ventricular septal occluders like the Amplatzer™, among others. In the past years, there has been an increase in the development of these devices as a result of a rise in the number of indications for implantation, paired with the aging and more medically complex patient population. This has led to an increase in the incidence of cardiac device-related infections, one of the most feared and serious complications which is associated with significant morbidity, mortality and financial burden.

Accurate diagnosis of cardiac device-related infections is essential given the management implications which often involve removal of the infected device, removal of other prosthetic material and long-term antimicrobial therapy. Clinical and laboratory data are useful diagnostic tools but multimodality imaging is often necessary. The recently published 2020 European Heart Rhythm Association International Consensus document, which is endorsed by many expert societies, has recommended the use of multimodality imaging for the diagnosis of CIED infections (1). Some of these modalities include: (1) soft tissue ultrasound which can identify abnormal fluid collections and guide aspiration procedures, (2) transesophageal echocardiogram which can identify concomitant endocarditis with CIED infection, (3) computed tomography which can evaluate local and distant extension of disease as well as allow pre-procedural planning, (4) magnetic resonance imaging which provides information on local and embolic manifestation of disease, (5) positron emission tomography (PET) which can identify metabolically active foci and assist on the diagnosis of infection, and (6) tagged white blood cell scan which similarly to PET can evaluate areas of inflammation suggestive of infection (2).

In this article we will review the utility of multimodality imaging for the diagnosis of cardiac device-related infections using a number of clinical cases to demonstrate its practical value.

## CIED Infection

CIEDs include implantable cardioverter defibrillators (ICDs), permanent pacemakers (PPM), and biventricular pacemakers which provide cardiac resynchronization therapy with or without a defibrillator (CRTs). Recently, novel devices including leadless pacemaker, subcutaneous ICD (SC-ICD) and implantable loop recorders (ILR) have been developed in an attempt to decrease the risk of infection but are not protected from infection. CIED infections are typically classified as pocket infections or systemic infections. Systemic infection may co-exist with cardiac valve infection, in which case the term CIED-related infective endocarditis (CIED-IE) is utilized (3).

### Pocket Infection

**Figure 1:**
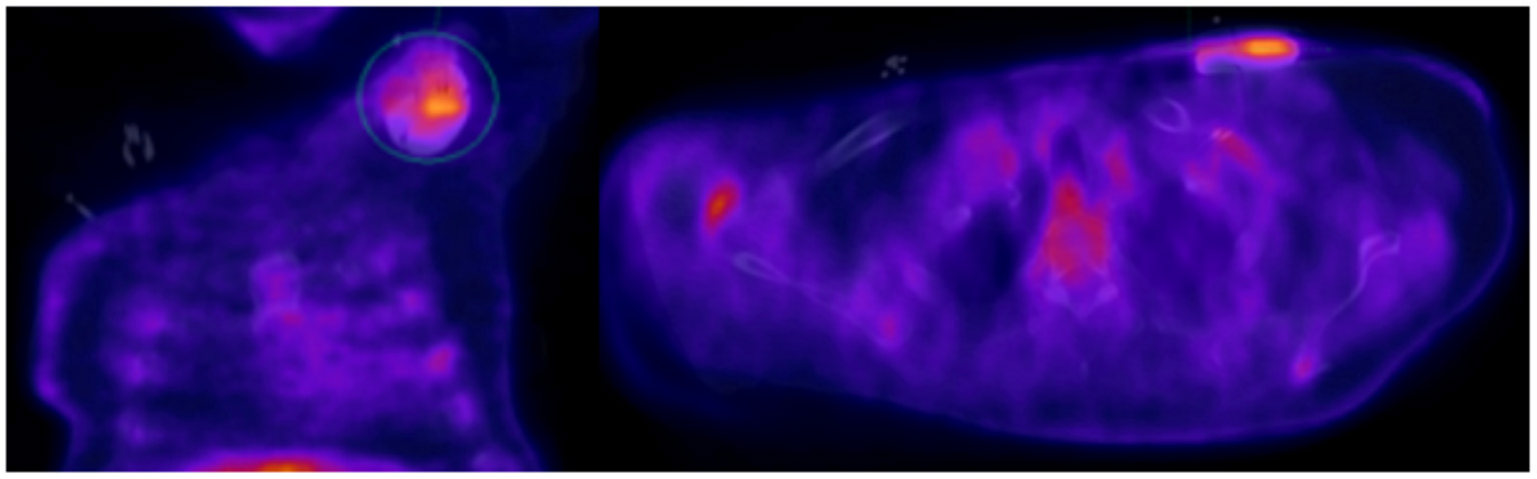
Seventy year-old male with sick sinus syndrome s/p dual chamber PPM 2 years prior, who presented with fever. PPM pocket site with mild tenderness on palpation but no obvious signs of infection. Infectious work up was unremarkable but given ongoing fever and high clinical suspicion for pocket infection, an ^18^F-FDG PET-CT was obtained showing increased FDG pocket uptake consistent with pocket infection. The patient underwent complete system removal.

Pocket infection is the most common type of CIED infection defined as infection limited to the generator pocket (4). Management typically involves entire CIED extraction (generator and transvenous leads) unless the infection is identified as early superficial site infection which can be managed with a course of antibiotics (5). Diagnosis of pocket infection is usually made clinically when there is presence of symptoms related to local inflammation. Soft tissue ultrasound can be done to assess for collections, although this is often not necessary. Pocket infection occasionally can be associated with CIED systemic infections and/or endocarditis (1). When the diagnosis pocket infection remains unclear or is equivocal, ^18^F-fluorodeoxyglucose positron emission tomography (^18^F-FDG PET) can provide important evidence. F18-FDG PET has a high diagnostic accuracy in the early diagnosis of pocket infection and is helpful in differentiating superficial from systemic infection. A recent meta-analysis showed very good sensitivity and specificity (93–96% and 97–98% respectively) for diagnosis of pocket infection which was reported to be higher than that for lead infection and endocarditis (6–8). The 2020 European Heart Rhythm Association International Consensus document recommend use of 18F-FDG PET-CT or WBC SPECT/CT in patients with positive blood cultures and clinically negative pocket and in patients with negative blood cultures with clinically negative pocket but high clinical suspicion (1) (Figure 1).

In cases where there is any concern of infection involvement beyond the superficial pocket site, as evidenced by positive blood cultures, systemic inflammatory response syndrome, purulent drainage and/or site erosion, one should suspect lead infection and further imaging work up with TTE and TEE is indicated to determine extent of infection (1, 9).

### CIED Systemic Infection With and Without IE

**Figure 2:**
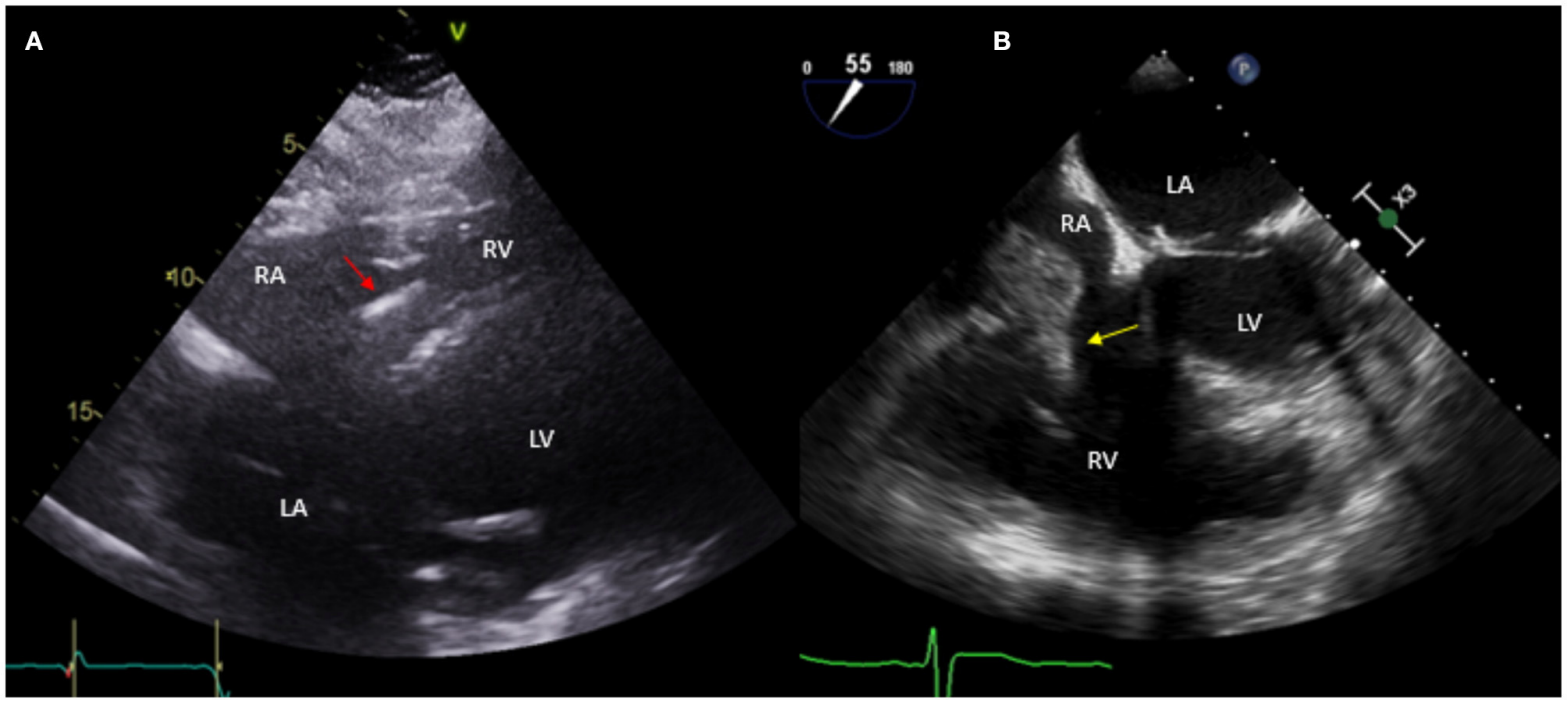
Fifty nine year-old male with a historyof ischemic cardiomyopathy (EF 35%) s/p ICD for primary prevention of sudden cardiac death who presented with fever and MSSE bacteremia. Initial TTE done **(A)** shows subcostal view with ICD lead in place (red arrow), without evident vegetations. Based on high clinical suspicion for CIED infection, a TEE was obtained **(B)** which showed a large vegetation attached to the ICD lead prolapsing into the right ventricle (yellow arrow). The patient was treated with IV antibiotics but given persistent bacteremia and large vegetation he underwent sternotomy with ICD system extraction and epicardial defibrillator placement.

The diagnosis of CIED systemic infection is suspected in the presence of unexplained fever, systemic symptoms, positive blood cultures and/or embolic phenomena (10). Diagnosis is typically confirmed with echocardiogram showing lead vegetations (1, 11). However, it is important to recognize that up to 13% of patients with CIED have asymptomatic lead masses, which often represent fibrin sheath and do not increase the risk of infection. Thus, the clinical presentation in such cases should be carefully evaluated and serial echocardiograms may be warranted (12). TEE has better sensitivity compared to TTE in detecting CIED infections (90 vs 22–43%). In addition, TEE allows better examination of the lead in the superior vena cava (1, 13–15) (Figure 2).

**Figure 3:**
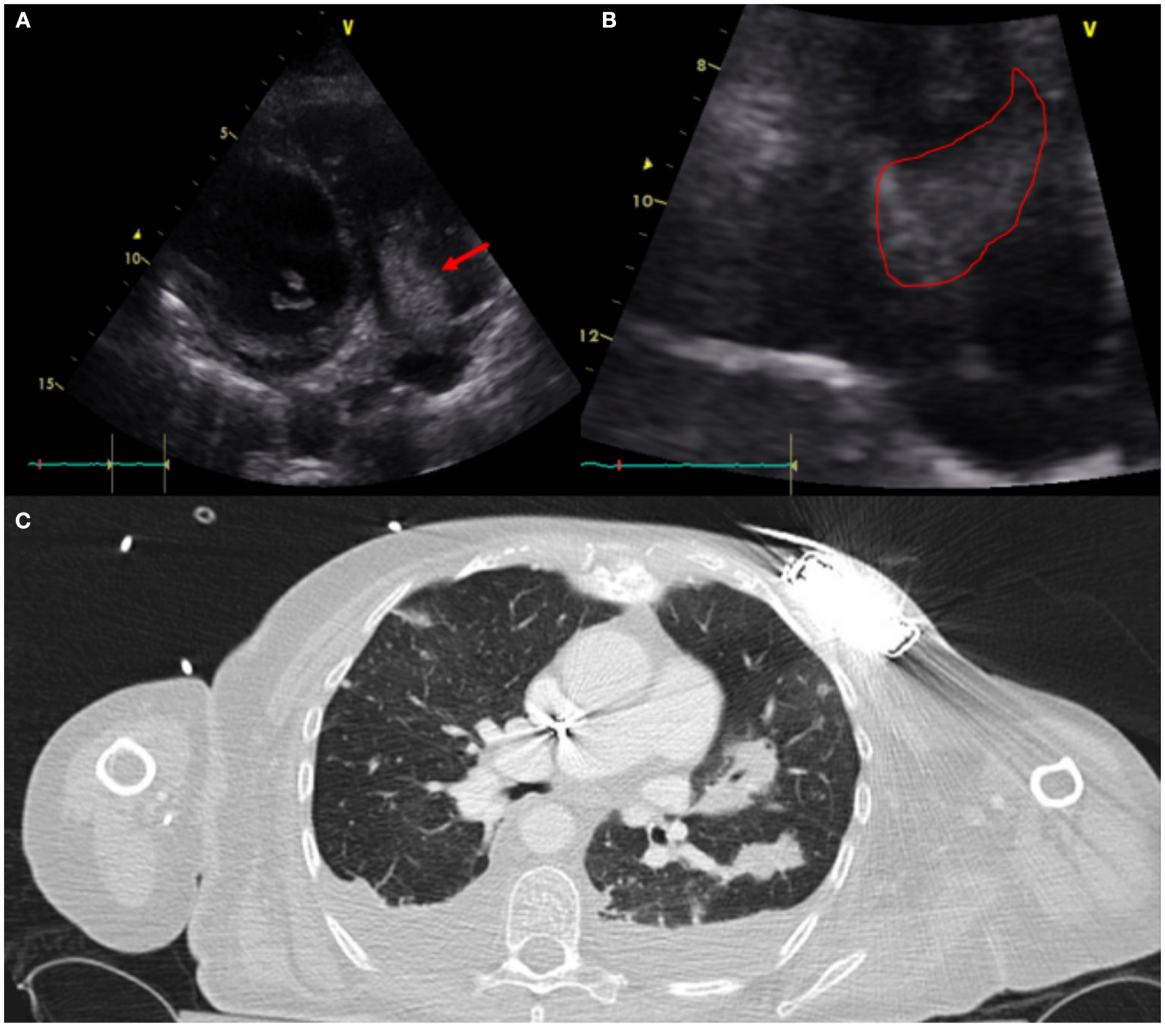
Sixty one year-old woman with a history of NICM s/p single lead ICD who presented with fever and malaise. Infectious work up was positive for MSSA bacteremia. TTE showed a very large vegetation attached to the ICD lead in the RA protruding into the RV [**(A)**-modified apical view and **(B)**-RV focused view with RA zoom]. A pre-procedural CT **(C)** was obtained and revealed bilateral, extensive and multifocal septic emboli with cavitary lesions as well as bilateral pleural effusions without evidence of pocket infection. The patient underwent open sternotomy with device extraction and TV repair for septal leaflet perforation for management of CIED systemic infection with IE.

CIED-IE most commonly affects the tricuspid valve (3, 16). This is particular important to diagnose because of its management implications that might require open heart surgery for system extraction and valvular intervention in some situations (i.e large >2cm vegetation and/or pulmonary embolism) versus percutaneoussystem extraction (5). In cases requiring system extraction only, the use of CT for pre-procedural planning has become a great asset to define adherence of leads to surrounding vasculature which would require more specialized equipment and more experienced operators for successful system extraction (17) (Figure 3).

**Figure 4:**
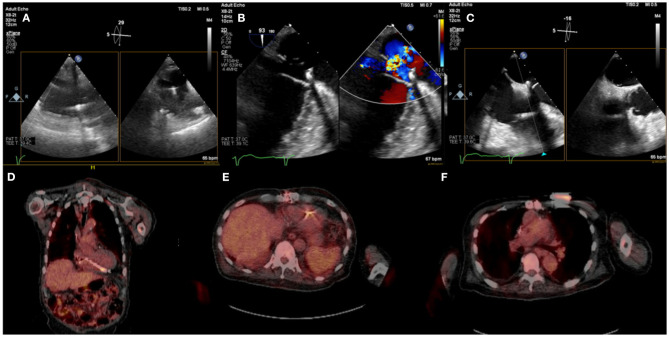
Eighty five year-old male with history ischemic cardiomyopathy s/p dual-chamber ICD for primary prevention, history of CABG and mitral valve repair presented with persistent MRSA bacteremia. TEE showed small linear echodensities attached to right atrial lead **(A)**, multiple small echodensities attached to mitral ring and moderate mitral regurgitation **(B)**. The RV showed no vegetations with normal tricuspid valve **(C)**. ^18^F-FDG PET-CT was obtained to assess extent of infection and showed focal uptake along the RV lead **(D,E)**. Left chest wall ICD showed focal uptake but no pocket uptake to suggest pocket infection **(F)**. The patient was deemed to have a prohibitive risk for redo cardiac surgery and was treated medically. He passed away 2 months after the diagnosis of CIED systemic infection with MRSA endocarditis.

In a small percentage of patients with suspected infection and non-diagnostic TEE or when TEE cannot be performed, alternative imaging methods are indicated (5, 11). A functional approach based on nuclear imaging using ^18^F-FDG PET-CT or radiolabeled WBC scintigraphy, has been incorporated to the 2015 ESC guidelines for management of endocarditis and the most recent 2020 European Heart Rhythm Association consensus for management of CIED infections (1, 18). The performance of ^18^F-FDG PET-CT in CIED infections was studied in a meta analysis by Juneau et al. and showed its high diagnostic accuracy with 87% sensitivity and 95% specificity for the diagnosis of IE (6). In addition, ^18^F-FDG PET-CT has the advantage of allowing imaging of multiple sites of possible infection (pocket/generator, leads) in one examination with a high diagnostic yield for septic emboli to the lungs or very rarely the bone structures which has management implications (i.e. need for longer antibiotic course with good bone penetrance in spondylodiscititis) (19, 20) (Figure 4).

Caution must be used when interpreting ^18^F-FDG PET-CT in early CIEDs (<2 months) as physiologic FDG uptake can be present without the presence of infection. Other disadvantages of using ^18^F-FDG PET-CT are high cost, not widely available, radiation, patient preparation and need for trained personal.

## Subcutaneous ICD (S-ICD)

The S-ICD involves no hardware exposure to the intravascular system. Pocket infections are rare, found in 1.7% of patients. Systemic infections are extremely rare (21, 22). Given its recent development, S-ICD infections have limited management data with only a few case reports found in the literature (23). Management is suggested in an individual basis based on extent of infection in which case these have been managed in a similar way to traditional pocket infections.

## Long-Term Mechanical Circulatory Support Devices Infection

Continuous flow ventricular support devices such as left ventricular assist devices (LVADs) and less frequently biventricular assist devices (BIVADs) are increasingly used for management of end-stage heart failure as bridge to transplantation (BTT) or destination therapy (DT) (24). As in CIEDs infection, LVADs infection is a serious complication that often involves prolong hospitalization, extended antibiotic and sometimes device extraction. For continuous-flow LVADs, infection occurs in 19–39% of patients and results in >10% of LVAD-related deaths (25).

LVAD infections are classified into two groups: LVAD-specific infections such as pump pocket infection (PPI), cannula infection and driveline infection (DLI); and VAD-related infections (endocarditis, bloodstream infection and mediastinitis). Diagnostic work up of VAD-specific and VAD-related infections remains challenging given lack of randomized data. Thus, current recommendations are based on expert consensus as published in the International Society of Heart and Lung Transplantation (ISHLT) guidelines for identification and management of LVADs infection (26).

LVAD DLI is the most common LVAD-specific infection because the driveline exit creates an entry tract for bacteria that can attach to prosthetic material with bacterial biofilm. DLI is sub-divided into deep and superficial. Both types involve soft tissue surrounding the driveline exit, whereas deep infections also involve the fascia and muscle layers (27). No imaging modality can definitely exclude deep tissue infection, thus imaging in diagnosis of LVAD infections remains a challenge and is based mostly on observational data (28). Soft tissue ultrasound can help identify fluid collections around the driveline but cannot rule out deeper infections. TTE and TEE are often not useful in this setting unless concomitant endocarditis is suspected. CT is commonly used given the ability to detect deeper DLI demonstrated by the presence of abscess and fat stranding but can be limited by metal device artifact. The sensitivity and specificity of CT has not yet been established.

**Figure 5:**
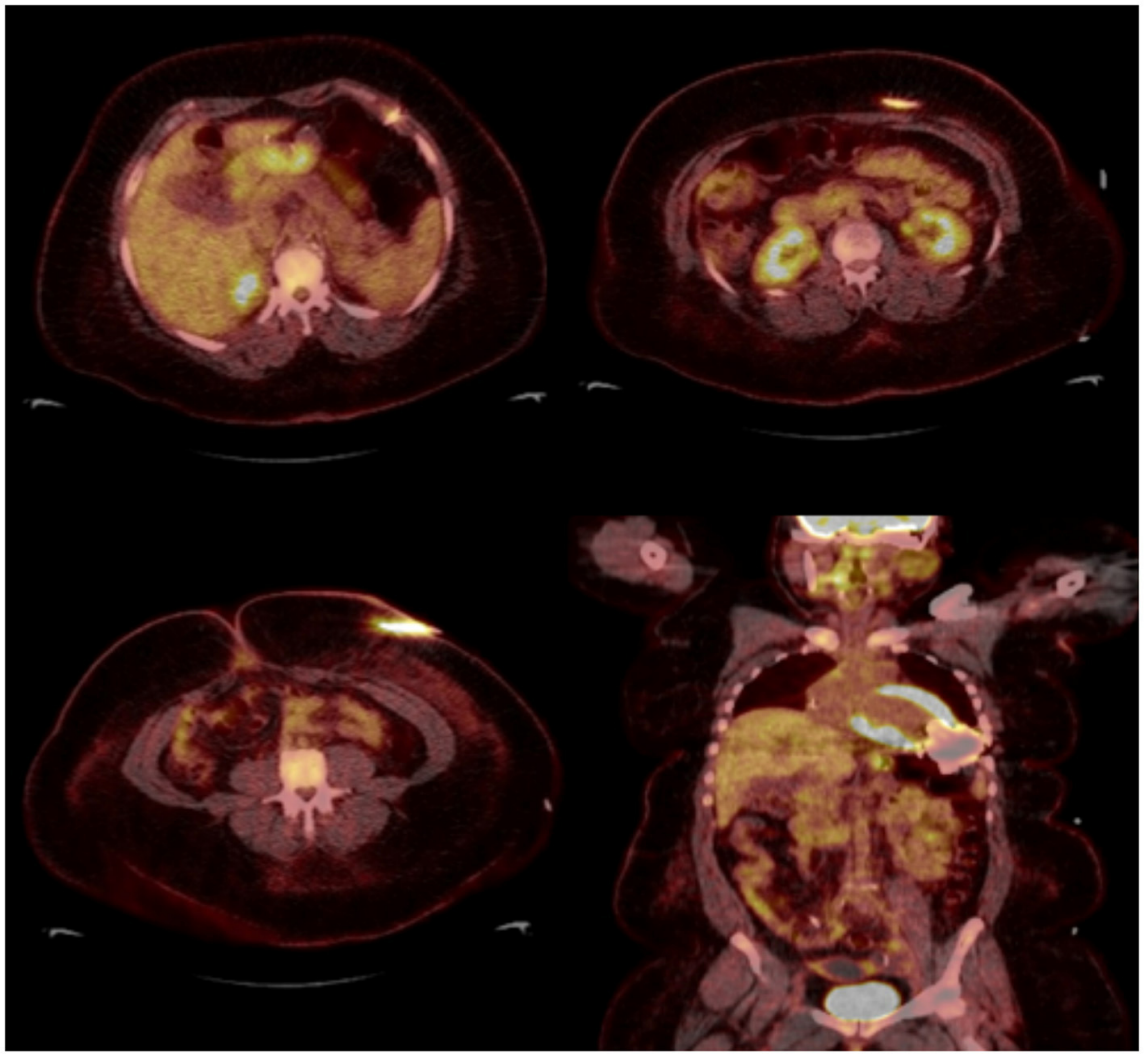
Thirty eight year-old female with end-stage heart failure s/p LVAD (Heart Mate 3) presented with drainage around driveline exit site and abdominal pain. She was treated with oral antibiotics for several weeks but continued to have symptoms. ^18^F-FDG PET-CT was obtained to assess extent of infection and showed increase FDG uptake surrounding LVAD driveline in the anterior abdominal wall without collections. She underwent driveline debridement for DLI and the driveline exit was relocated medially.

**Figure 6:**
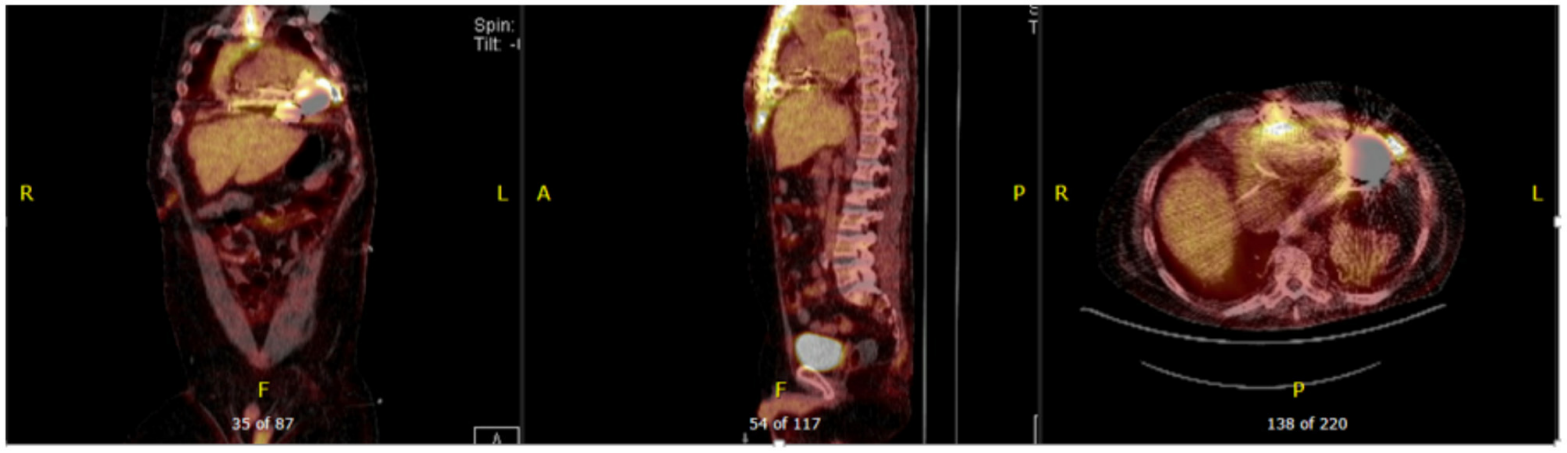
Forty seven year-old male with ischemic cardiomyopathy s/p LVAD (Heart Mate 3) presented with suspected DLI, with wound cultures positive for Candida Albicans. ^18^F-FDG PET-CT showed FDG activity in the pericardium, mediastinum, surrounding LVAD pump, driveline and sternum consistent with deep and superficial LVAD infection. He was managed with systemic lifelong antifungal therapy.

^18^F-FDG PET-CT and tagged WBC scan have emerged as more accurate modalities for detection of LVAD infection based on mostly small non-randomized studies (29–31). Correlating the anatomic findings, for example an abscess with the metabolic information is particularly useful. A major limitation of ^18^F-FDG PET-CT is the presence of non-specific uptake that can be present not only in early post-cardiac surgery but even years after LVAD implantation described as a post-operative inflammatory response. This tends to be characterized by a lesser degree of uptake and the reading clinician should become familiar with this presentation. Another consideration is the normal uptake pattern in patients with prosthetic materials due to foreign-body chronic low-grade inflammation. This is frequently seen when Dacron material is present in the LVAD outflow. Vaugelade et al. considered that a circumferential and homogeneous ^18^F-FDG uptake was a negative pattern for infection, defining positivity as a focal ^18^F-FDG uptake observed on both attenuation-corrected and uncorrected images in order to avoid overcorrection artifacts (31) (Figures 5, 6).

## LAAO Device Infection (Watchman™ Infection)

**Figure 7:**
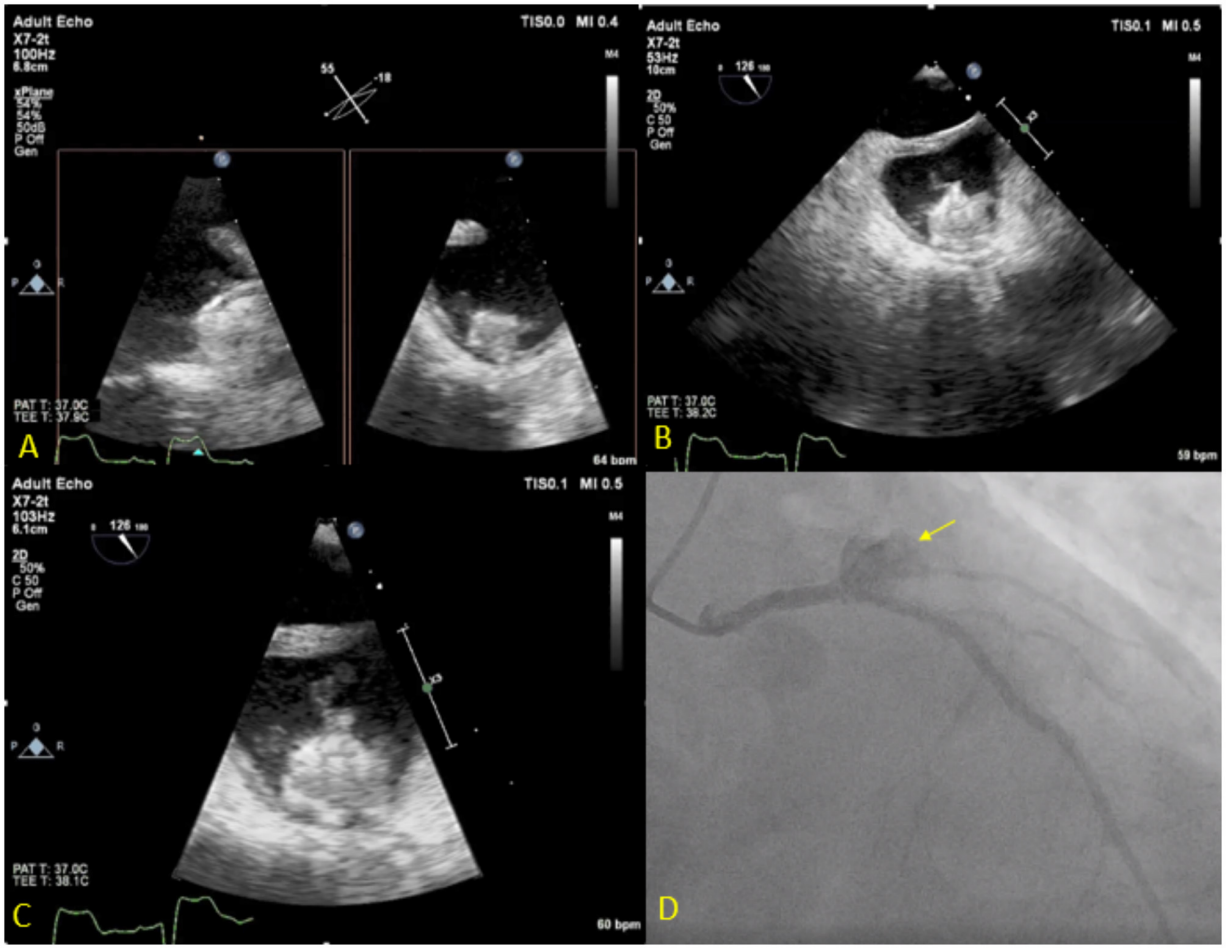
Seventy three year-old male with history of CAD s/p multiple PCIs and Afib s/p Watchman implantation 3 weeks prior to presentation, presented with fever, chest pain and confusion. Blood cultures grew Serratiamarcensens. TEE **(A–C)** shows Watchman device in the left atrial appendage with large mobile vegetations. Course was complicated by distal embolization to the brain an intestine vasculature. Patient developed worsening chest pain and ST depressions, coronary angiogram **(D)** shows total occlusion of the proximal left circumflex artery secondary to mycotic aneurysm (Arrow). The patient was deemed too high risk for surgery and was treated medically.

Watchman™ device infection is very rare, with only a few cases reported in the literature (32). The incidence is estimated to be much lower than other CIED infections given that endothelization of the device is expected within 45 days of implantation. However lack of endothelization has been described and this may be associated with higher risk of infection (33). The approach to Watchman^®^ device infection should be based on current infective endocarditis guidelines; which includes obtaining blood cultures and TEE when there is suspicion for infection (Figure 7).

## Septal Occluder Infection

Infection of septal occluder devices is uncommon and reported as the least common complications of this procedure. Like with LAAO occlusion, only case report literature is available with as little as 3 cases of septal occluder device infection reported in the literature (34). Infection of these devices has been classified as early (within 6 months of implantation, prior to endothelization) which are thought to be secondary to introduction of microorganisms during the initial procedure; and late, when infection occurs secondary to seeding of microorganisms from distant sources like in IE. In the presence of infection involving septal occluder devices, prolonged antimicrobial therapy with constant blood culture monitoring has been reported to be adequate in the absence of device dehiscence, septal perforation or fistula formation.

## Conclusion

The use of cardiac implantable electronic devices is increasing. These include permanent pacemakers and implantable cardiac defibrillators, ventricular assistance devices (VADs), left atrial appendage occlusion (LAAO) devices, atrial and ventricular septal occluders, among others. Despite its overall safety profile, CIED infection can occur and is associated with significant morbidity, mortality and financial burden. Multimodality imaging including TTE, TEE, CT, ^18^F-FDG PET-CT and tagged WBC can assist in establishing a diagnosis, guiding therapy and providing the patient and physician with prognostic information.

## Author Contributions

JA contributed in writing manuscript and collecting images. EH and WJ contributed in reviewing and making changes in manuscript and also collecting images for cases. All authors contributed to manuscript revision, read, and approved the submitted version.

## Conflict of Interest

The authors declare that the research was conducted in the absence of any commercial or financial relationships that could be construed as a potential conflict of interest.

## Publisher's Note

All claims expressed in this article are solely those of the authors and do not necessarily represent those of their affiliated organizations, or those of the publisher, the editors and the reviewers. Any product that may be evaluated in this article, or claim that may be made by its manufacturer, is not guaranteed or endorsed by the publisher.
